# Weight loss increases all-cause mortality in overweight or obese patients with diabetes: A meta-analysis

**DOI:** 10.1097/MD.0000000000012075

**Published:** 2018-08-21

**Authors:** Yiqi Chen, Xue Yang, Juyang Wang, Yangshiyu Li, Dou Ying, Huijuan Yuan

**Affiliations:** Department of Endocrinology and Metabolism, Diabetes Research Center, Henan Provincial People's Hospital, Zhengzhou, HeNan Province, China.

**Keywords:** diabetes, mortality, obese, overweight, weight loss

## Abstract

**Background:**

Overweight and obese patients with diabetes are advised to lose weight to improve their health; however, recent studies have demonstrated that weight loss may be associated with worse long-term survival in patients with diabetes. This meta-analysis aimed to examine the relationships between weight loss and all-cause mortality in overweight or obese individuals with diabetes.

**Methods:**

We searched the PubMed and EMBASE databases from inception to February 2017. We included prospective studies that reported sufficient information to extract mortality-specific relative risks (RRs) with corresponding 95% confidence intervals (CIs). RRs with 95% CIs were pooled using a random-effects model. A subgroup analysis was also performed to explore sources of heterogeneity.

**Results:**

Of the 1652 studies identified, 8 met the inclusion criteria. A total of 18,887 patients were included in this analysis. We found that compared with a stable weight, weight loss was associated with an increased risk of all-cause mortality (RR, 1.15; 95% CI, 1.04 to 1.28) and cardiovascular disease (CVD) mortality (RR, 1.15; 95% CI, 1.02 to 1.29) in overweight or obese adults with diabetes, whereas intentional weight loss was not associated with changes in all-cause mortality (RR, 0.90; 95% CI, 0.67 to 1.22). Weight gain was not associated with changes in all-cause mortality (RR, 1.17; 95% CI, 0.87 to 1.58) or CVD mortality (RR, 0.97; 95% CI, 0.93 to 1.01). Compared with an initial body mass index (BMI) of 25 to 30 kg/m^2^, an initial BMI of >35 kg/m^2^ was associated with increased all-cause mortality (RR, 1.23; 95% CI, 1.01 to 1.50), which was further increased with an initial BMI of >40 kg/m^2^ (RR, 1.50; 95% CI, 1.16 to 1.94).

**Conclusion:**

Our results indicate that weight loss but not weight gain increased all-cause mortality and CVD mortality in overweight or obese adults with diabetes.

## Abstract 2

Supplemental Digital Content is available in the text

## 1 Introduction

In the US, approximately 45% to 65% of patients with type 2 diabetes mellitus (T2DM) are obese.^[1]^ Overweight and obese patients with diabetes mellitus (DM) are advised to lose weight not only to improve their glycemic control, quality of life, mobility, and physical functionality but also to reduce their cardiovascular risk factors, medications required to manage diabetes and long-term healthcare costs.^[2]^ Moreover, a recent meta-analysis of data from approximately 20,000 participants concluded that compared with weight stability, an intentional weight loss of 5.5 kg in obese adults was associated with an approximately 15% reduction in all-cause mortality.^[3]^

However, these conclusions are not uniformly accepted. Specifically, weight loss may be a notably significant indicator for the development of life-threatening, systemic illness, such as cancer.^[4–6]^ Several studies that suggested that weight loss appeared to be associated with worse long-term survival in patients with diabetes^[7]^ have further complicated this debate. While the reasons for this association are unclear, they may be rooted in the obesity paradox. One study included 10,568 patients with diabetes who were followed for a median of 10.6 years. The results indicated that being overweight was associated with a lower mortality risk, whereas obese patients had a mortality risk similar to that of normal-weight individuals.^[8]^ Consequently, this set of controversial findings casts doubt on current clinical practice guidelines and leaves clinicians with substantial uncertainty regarding the value of weight loss in patients with diabetes. The aim of the present study was to examine the impact of weight loss on all-cause mortality in overweight or obese adults with diabetes and to explore possible reasons for these conflicting results.

## 2 Materials and methods

### 2.1 Literature search

The PubMed and EMBASE electronic databases were searched from inception to February 2017 to identify relevant studies. We used a combination of keywords related to the types of weight loss, diabetes, and mortality. The syntax used for Medline is provided in Table 1. The search strategies used for the other databases were similar, with the necessary adaptations employed. An English language restriction was imposed. We also evaluated the references in the pertinent review articles and meta-analyses to identify other potentially eligible studies. The methodological quality of the studies was evaluated using the Newcastle–Ottawa scale.^[9]^ The maximum Newcastle–Ottawa scale score is 9: quality of selection (maximum, 4 stars), comparability (maximum, 2 stars), and exposure (maximum, 3 stars). A high-quality study was defined as a score equal to or greater than 7, and we defined a score from 4 to 6 as a moderate-quality study. All aspects of the study comply with the Declaration of Helsinki and the study was approved by the Ethics Committee of the Zhengzhou University People's Hospital.

**Table 1:**
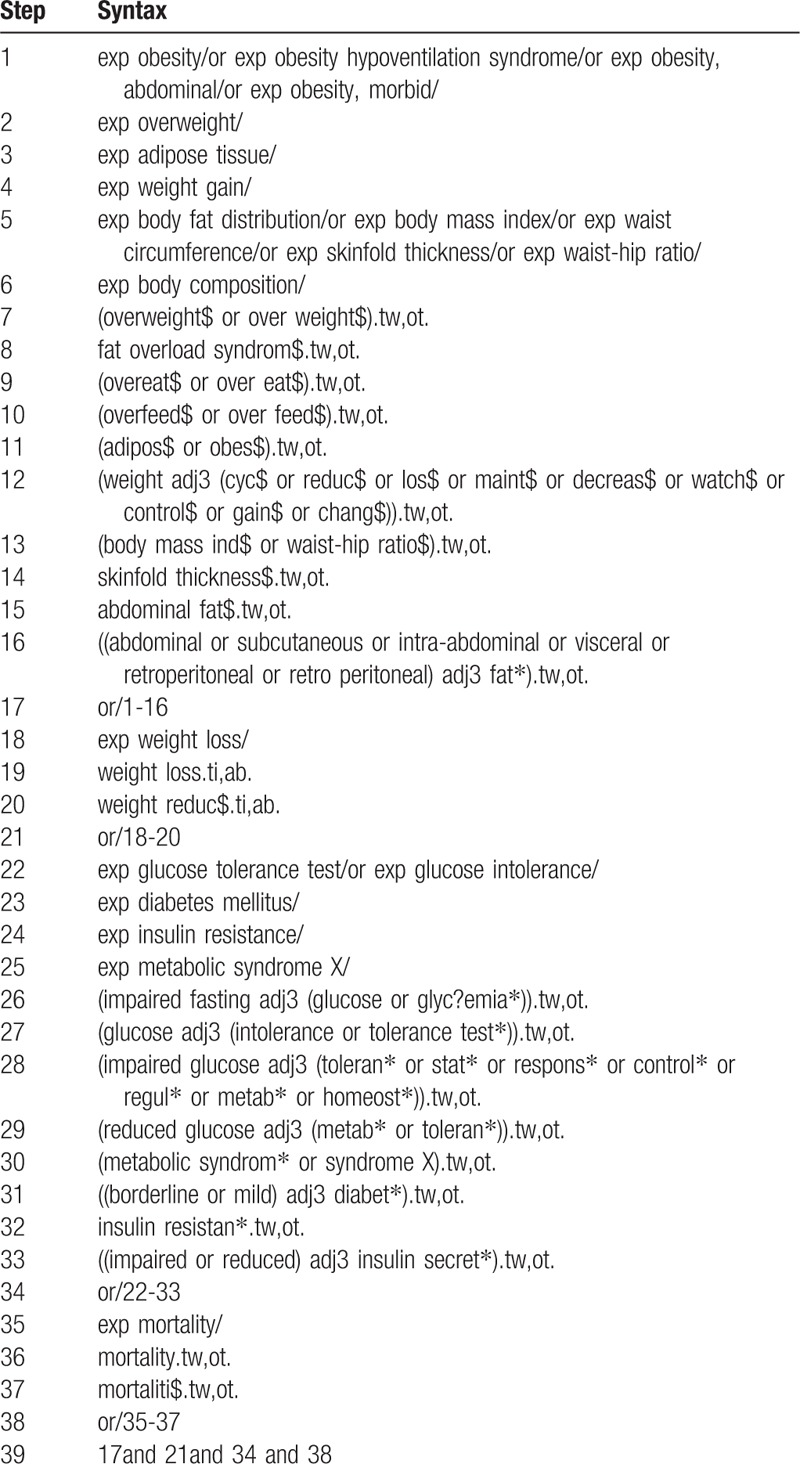
Search strategy for Medline.

### 2.2 Inclusion criteria

A randomized controlled trial or an observational study was included if it met the following criteria: the weight changes in adult patients who were diagnosed with or self-reported DM were compared with those in controls; and adjusted and unadjusted mortality data were available.

A published study was included if it included patients with clinical DM, measured weight losses/changes, compared weight-loss and control groups, had a follow-up period ≥2 years, and reported the adjusted effect size and its 95% confidence interval (CI). In cases of duplicate publications, we only included the most informative and complete studies. We did not include editorial letters, systematic reviews, meta-analyses, conference abstracts, and commentaries. Studies were deemed suitable only if they included full details of the statistical models, including the confounding factors. A list of the excluded studies and reasons for exclusion is provided in the table in Appendix 1;.

### 2.3 Data extraction and quality assessment

Data were extracted independently by 2 investigators (HJY and YQC) in May 2016. Discrepancies were resolved by consensus or according to the third author's (XY) judgment. The search was repeated in February 2017 to identify any additional studies meeting the inclusion criteria. Authors were contacted in person, if required, to obtain further details regarding articles that met inclusion criteria. The results for each study were extracted for maximally adjusted models. We extracted the following data from each study: the first author's name, publication year, study period, country or region where the study was conducted, sample size, weight change definition, the average participant age, the type of patients, initial BMI, diabetes duration, the manner of losing weight, comparison group, risk ratios (RRs) or hazard ratios (HRs) and 95% CIs for weight change categories, and variables adjusted for in the analysis (Tables 2–7).

**Table 2:**
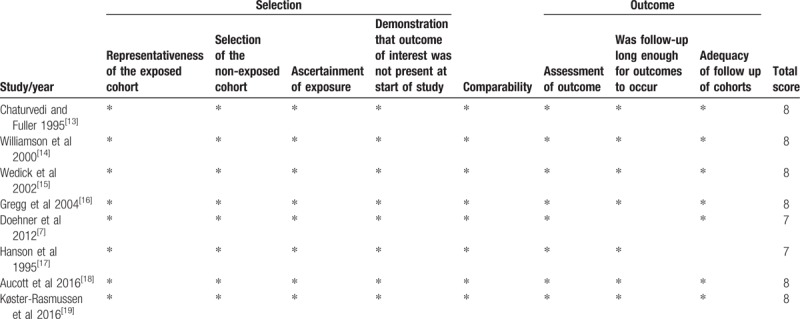
Methodological quality assessment (risk of bias) of the included studies according to the Newcastle–Ottawa scale.

**Table 3:**
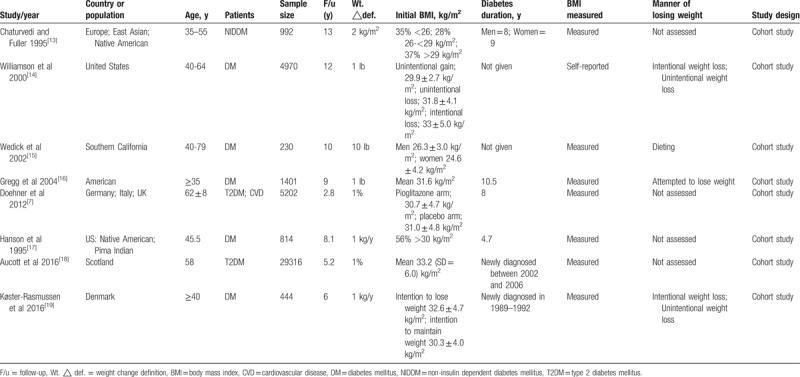
Included studies and characteristics.

**Table 4:**
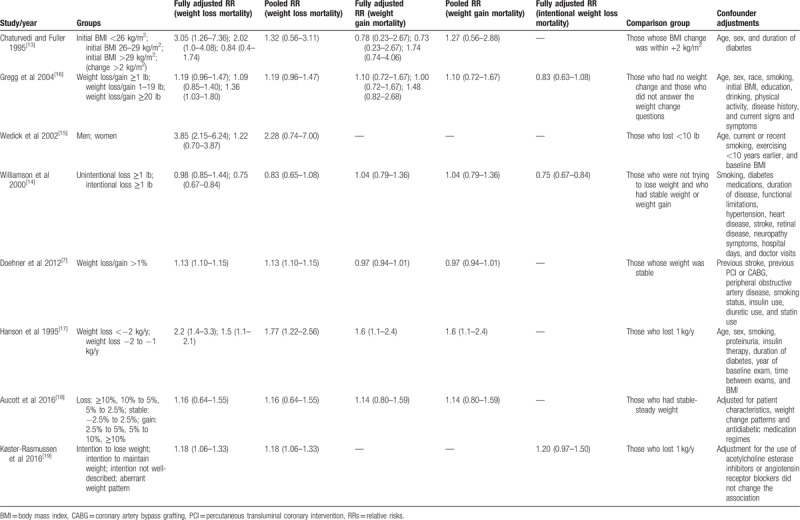
Relative risks for all-cause mortality associated with weight change in overweight or obese individuals with diabetes.

**Table 5:**
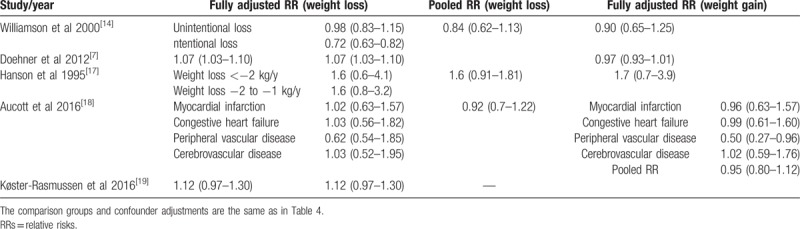
Relative risk for cardiovascular disease mortality associated with weight change in overweight or obese individuals with diabetes.

**Table 6:**
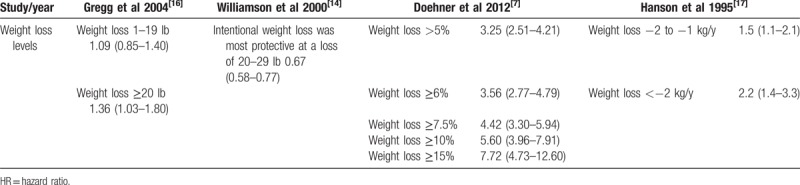
Hazard ratios for comparison between different levels of weight loss in all-cause mortality in overweight or obese individuals with diabetes: weight stable as reference group.

**Table 7:**
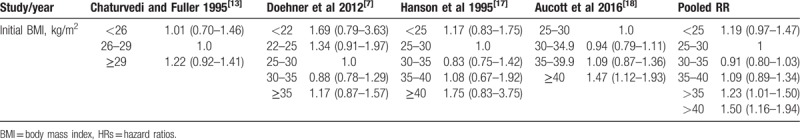
Hazard ratio for comparison between body mass index categories to predict all-cause mortality in overweight or obese individuals with diabetes: initial body mass index category 25 to 30 kg/m^2^ as reference group.

### 2.4 Data analysis

In the examination of the associations of weight loss with all-cause mortality in overweight or obese diabetic individuals, the results are expressed as RRs with 95% CIs; RRs and HRs were included as eligible RRs without distinction because each provided effect sizes of a similar magnitude.^[10]^ Extracted HRs were recalibrated if the reference group was not weight stable. For example, if an article reported results for 4 different categories—such as weight stable-steady, weight stable-cyclic, weight gain, weight loss—and their reference category was weight loss, then the HRs would be recalibrated so that the weight stable-steady group would be the reference category. Extracted HRs were pooled when HRs for different groups were reported. For example, if an article reported results for 2 different categories, such as, weight loss HRs for men and women with diabetes, we pooled the HRs for all the participants with diabetes. Heterogeneity was assessed using the *I*^2^ statistic; *P* values <.05 were considered significant.^[9]^*I*^2^ illustrates the proportion of total variability attributed to between-study variation; *I*^2^ values of 25%, 50%, and 75% represent low, moderate, and high heterogeneity, respectively.^[11]^ Thus, we determined multivariate-adjusted RRs with 95% CIs using a random-effects model. Statistical analysis was performed using Review Manager (RevMan) [Computer program]. Version 5.3. Copenhagen: The Nordic Cochrane Centre, The Cochrane Collaboration). We performed a meta-analysis by removing 1 outcome at a time to investigate whether each study contributed substantially to heterogeneity^[12]^ (Table 8). Publication bias was examined using Egger's regression test.

**Table 8:**
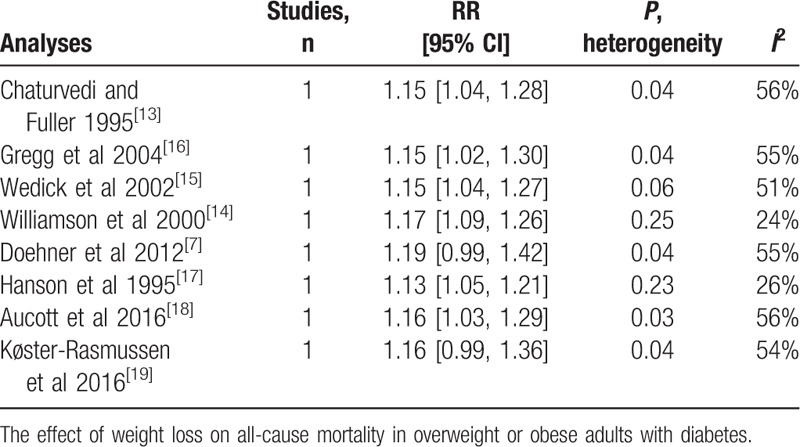
One-study-out method for sensitivity analysis.

## 3 Results

### 3.1 Study identification and selection

**Figure 1:**
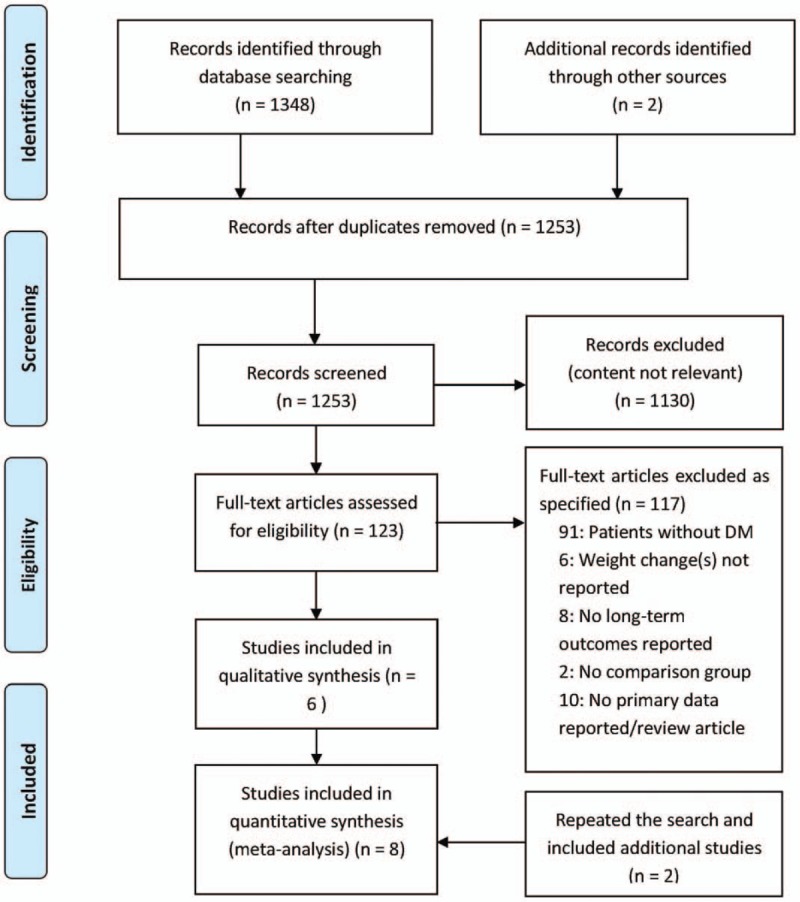
Figure legends.

A total of 1348 studies were identified in the primary search after the removal of duplicate studies in May 2016. In all, 121 full-text articles were reviewed, of which 91 were excluded because the participants were not diabetics, 6 were excluded because weight change (s) were not reported, 8 were excluded because no long-term outcomes were reported, 2 were excluded because a control group was lacking, and 10 were excluded because no primary data were reported or because the publication was a review article. A total of 6 studies were included in the analysis.^[7,13–17]^ The search was repeated in February 2017, which yielded 201 studies published from May 2016 to February 2017, of which 173 were excluded as unrelated, 13 were excluded because they were reviews and meta-analyses, 9 were excluded because the participants were not diabetics, and 4 were excluded because the results did not concern all-cause mortality. Two studies were added to the final analysis^[18,19]^ with a total of 8 studies included in the final analysis^[7,13–19]^ (Fig. 1).

### 3.2 Study characteristics

The included studies are summarized in Table 3. In total, these studies included 18,887 individuals with a mean follow-up period of 9.5 years. Two studies were conducted in the United States.^[14,16]^ One study was conducted in Germany, Italy, and the UK^[7]^; 1 study was conducted in Native American Pima Indians.^[17]^; 1 was conducted in Europeans, East Asians, and Native Americans^[13]^; and the remaining studies were conducted in southern California, Scotland or Denmark. The populations comprised middle-aged and older adults. In 2 studies, body weight and weight change were self-reported, which may cause high heterogeneity,^[7,14]^ and the remaining 6 studies measured weight and height at all visits. Tables 4 and 5 provide details on all-cause and cardiovascular mortality for each study and the estimates and corresponding 95% CIs extracted for each weight change category. All studies were cohort studies. The NOS results are shown in Table 2. The 8 included studies were all of high quality.

### 3.3 Weight loss

**Figure 2:**
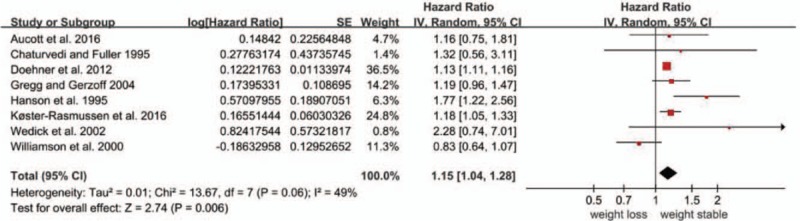
Forest plot showing the effect of weight loss on all-cause mortality.

All 8 studies examined the relationship between weight loss and all-cause mortality in overweight or obese individuals with diabetes. The overall pooled relative risk of all-cause mortality for the weight loss group was 1.15 (95% CI, 1.04 to 1.28) (Fig. 2), which indicates that weight loss increased all-cause mortality in overweight or obese patients with diabetes. Moderate heterogeneity was significant among the estimates reported by the included studies (Q test, *P* = 0.06, *I*^2^ = 49%). When the 2 studies in which weight change was self-reported were omitted, the result was 1.24 (95% CI, 1.10 to 1.39), and a significant decrease in heterogeneity was observed (Q test, *P* = 0.36, *I*^2^ = 9%). Omitting 1 study at a time did not substantially change the overall results (Table 8).

Regarding the effect of the degree of weight loss on all-cause mortality in overweight or obese patients with diabetes, 4 studies had pertinent results^[7,14,16,17]^; however, the studies used different measurements, the results of which are summarized in Table 6. As shown in this table, we found that the greater the weight loss, the greater the all-cause mortality.

The effect of weight loss on all-cause mortality differed for the category of initial BMI (Table 7). When the initial BMI was greater than 35 kg/m^2^, weight loss was associated with increased all-cause mortality.^[7,13,17,18]^

**Figure 3:**
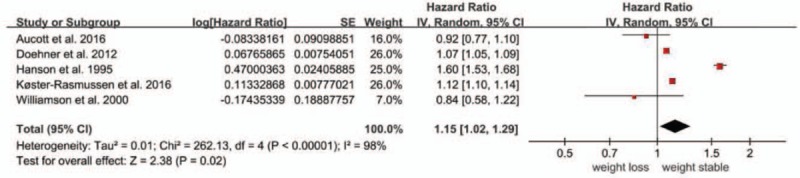
Forest plot showing the effect of weight loss on cardiovascular disease (CVD) mortality.

**Figure 4:**
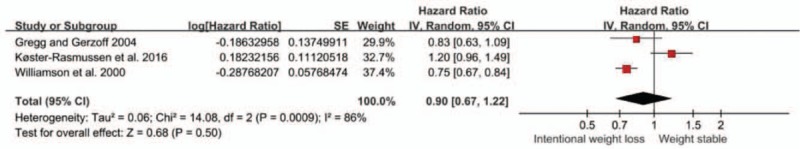
Forest plot showing the effect of intentional weight loss on all-cause mortality.

Five studies assessed the association between weight loss and cardiovascular disease (CVD) mortality^[7,14,17–19]^ and 3 assessed that between intentional weight loss and all-cause mortality.^[14,16,18]^ Compared to the reference group, an increased risk of CVD mortality was observed (HR, 1.15; 95% CI, 1.02 to 1.29), whereas intentional weight loss was not associated with all-cause mortality (HR, 0.90; 95% CI, 0.67 to 1.22). Notably, heterogeneity existed for CVD mortality (Q test, *P* < 0.001, *I*^2^ = 98%), and all-cause mortality of intentional weight loss (Q test, *P* < 0.001, *I*^2^ = 86%) (Figs. 3 and 4).

### 3.4 Weight gain

**Figure 5:**
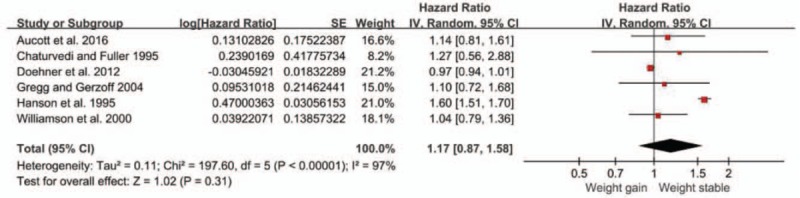
Forest plot showing the effect of weight gain on all-cause mortality.

A total of 6 studies investigated the association between weight gain and all-cause mortality.^[7,13,14,16–18]^ The overall pooled relative risk of all-cause mortality for the weight gain group was 1.17 (95% CI, 0.87 to 1.58; *P* = 0.31) (Fig. 5), which signified that weight gain was not associated with all-cause mortality in overweight or obese patients with diabetes. High heterogeneity was significant among the estimates reported by the included studies (Q test, *P* < 0.01, *I*^2^ = 97%). Omitting 1 study at a time did not substantially change the overall results. However, when 1 study was omitted, the heterogeneity decreased significantly (Q test, *P* = 0.77, *I*^2^ = 0%).

**Figure 6:**
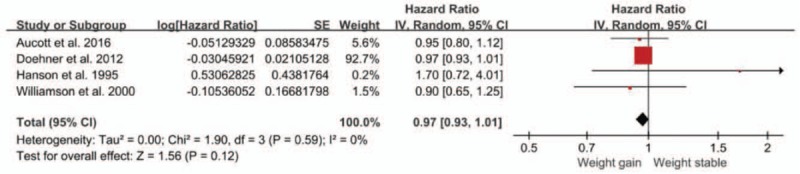
Forest plot showing the effect of weight gain on cardiovascular disease (CVD) mortality.

Four studies assessed the association between weight gain and CVD mortality.^[7,14,17,18]^ The overall pooled relative risk of CVD mortality for the weight gain group was 0.97 (95% CI, 0.93 to 1.01) (Fig. 6), which indicates that weight gain was not associated with CVD mortality in overweight or obese patients with diabetes. No heterogeneity existed among the estimates reported by the included studies (Q test, *P* = 0.59, *I*^2^ = 0%). No publication bias was identified using Egger's test (*P* = 0.557).

## 4 Discussion

To the best of our knowledge, this is the first meta-analysis to explore the effect of weight change on all-cause mortality in adult patients with diabetes. This study suggests that weight loss increased all-cause and CVD mortality in overweight or obese patients with diabetes, whereas weight gain was also not associated with all-cause and CVD mortality.

“The obesity paradox” suggests that the relationship between excess adiposity and mortality is unclear, with recent data suggesting that individuals who have a normal weight at the time of DM diagnosis may have a greater mortality risk than their overweight or obese counterparts.^[1]^ Thus, weight loss may not be beneficial for overweight or obese individuals with diabetes.

People with T2DM have difficulty losing weight, for several reasons. In insulin-resistant conditions, hyperinsulinemia promotes triglyceride synthesis and storage while inhibiting lipolysis in adipocytes, resulting in an expansion of adipose tissue.^[20]^ People with diabetes may live sedentary lifestyles and not be very physically active.^[21]^ Moreover, some of the commonly used glucose-lowering drugs, such as insulin and the sulfonylurea drugs, are associated with weight gain, which further complicates successful weight management.^[22]^ The diligent control of blood glucose rather than weight loss might benefit diabetes and decrease cardiovascular risk factors.^[23]^ Finally, weight regain may result from the compensatory response to hormonal and metabolic changes following initial weight loss, wherein orexigenic mediators that stimulate appetite persist.^[24]^ Therefore, weight loss is an abnormal phenomenon and this observation is noteworthy because weight loss may indicate poorly controlled plasma glucose, more severe disease at baseline, or perhaps an occult systemic illness (i.e., malignancy) that manifested itself later in the disease course and resulted in harmful weight loss.^[25]^

Other reasons might explain the association of weight loss with increased all-cause mortality in overweight or obese individuals with diabetes. First, our sample was composed of middle-aged and older adults. Muscle mass decreases with age and concurrently, fat mass, particularly the proportion of visceral and abdominal fat, increases. Moreover, weight loss via energy restriction may do little to alter the relative distribution of body fat and may result in decreased muscle mass, which is harmful for middle-aged and older adults.^[26]^ Second, diabetic patients had significantly higher scores for depressed mood than those without DM.^[27]^ Depression has been linked to weight loss and mortality.^[28]^ Third, some studies suggested that 1 possible explanation for the association of weight loss and higher mortality was occult disease.^[25]^

Our study also found that intentional weight loss did not increase all-cause mortality in overweight or obese adults with diabetes. In a recent meta-analysis, Harrington found an increased risk of all-cause mortality regardless of the intentional or unintentional nature of the weight loss among healthy participants; however, intentional weight loss had a small benefit for individuals classified as unhealthy (i.e., with obesity-related risk factors).^[29]^ The Look AHEAD Research Group found that intensive lifestyle intervention also produced greater reductions in hemoglobin A1c and greater initial improvements in fitness and all cardiovascular risk factors, except LDL cholesterol.^[30]^ Intentional weight loss may benefit diabetic patients for 2 possible reasons. First, patients who intend to lose weight may become more motivated to make a series of lifestyle changes, such as reducing their fat intake or increasing their exercise level. Such changes may decrease mortality by benefiting an individual's overall health status.^[30]^ Second, these individuals may become more likely to engage in positive health behaviors unrelated to weight (e.g., getting adequate sleep, not smoking), have more frequent contact with health care providers and participate in preventive care practices, such as early disease screening and treatment. However, only 3 such studies were included in the analysis, and high heterogeneity was observed (Q test, *P* < 0.001, *I*^2^ = 86%). In the future, well-designed studies that identify whether weight loss intention modifies the association between weight loss and all-cause mortality in overweight or obese patients with diabetes will be required to better understand the underlying pathways.

Our findings show that weight gain was not associated with changes in all-cause mortality or CVD mortality in overweight or obese adults with diabetes. The term “obesity paradox” has been coined to describe this association, which is supported by a large amount of data assembled from patients with DM or CVD showing that overweight and obesity are actually associated with prolonged survival.^[8,31]^ Conversely, weight loss is a characteristic feature of advanced illness, such as DM or CVD, and weight gain may therefore reflect reduce catabolic activity and restored anabolic capacity. In addition, some therapies, such as insulin, sulfonylurea drugs, ACE inhibitors and beta-blockers, are highly effective in improving survival in patients with DM or CVD and have been shown to produce weight gain. In spite of these data, our results indicate that all-cause mortality gradually increases when the initial BMI >35 kg/m^2^, suggesting that not all weight gain is associated with reduced mortality (Table 7). Thus, more research and date are needed to support the effect of weight gain on all-cause mortality in overweight or obese individuals with diabetes.

The studies included in this analysis had different inclusion criteria, such as the initial BMI, degree of weight change, sex, race, and weight-loss intent; for example, 5 different definitions of weight change were encountered, that is, a weight change of >2 kg/m^2^, 1 lb, 10 lb, 1% or 1 kg/y, and these differences may have contributed to the high heterogeneity observed. Two studies were conducted in the United States and the remaining studies were conducted in different countries. One of the studies included patients with T2DM and cardiovascular comorbidities, another included patients with T2DM only, and the remaining 4 studies included patients with DM; these differences may also have contributed to the high heterogeneity. When the study that included patients with T2DM and cardiovascular co-morbidities was omitted, the results were as follows: RR, 1.16; 95% CI, 1.06 to 1.27; *I*^2^ = 55%; *P* = 0.001.

A well-designed prospective study is necessary to conclusively determine the importance of weight loss in patients with established DM. Such a study should be adequately powered for long-term outcomes and should carefully assess body composition changes, use behavioral weight-loss strategies, encourage high-calorie expenditure exercise (e.g., walking frequently, walking long distances), and carefully control for cancer development and smoking cessation. Such a study would provide more conclusive evidence of the effect of intentional weight loss on the prognosis of patients with diabetes, answer multiple remaining questions, increase confidence in weight-management recommendations for patients with diabetes, and further clarify the obesity paradox. Additionally, given the known benefits of bariatric surgery in the general population, a randomized controlled trial of bariatric surgery in patients with diabetes might also be appropriate.

### 4.1 Limitations of the study

This meta-analysis was limited by the small number of studies included and the sample sizes, both of which increased the difficulty in performing subgroup analyses. Another potential limitation was that weight was measured directly in some studies and was self-reported in others.

## 5 Conclusion

Considering the limitations of this analysis, the evidence suggested that weight loss but not weight gain increased all-cause mortality and CVD mortality in overweight or obese patients with diabetes. In the future, studies using larger sample sizes and more accurate measurements of weight will be required to examine the relationship between weight loss and all-cause mortality in overweight or obese patients with diabetes.

## Author contributions

**Conceptualization:** Yangshiyu Li, Huijuan Yuan.

**Data curation:** Yiqi Chen, Huijuan Yuan.

**Formal analysis:** Yiqi Chen, Yangshiyu Li, Huijuan Yuan.

**Investigation:** Yiqi Chen, Juyang Wang.

**Methodology:** Yiqi Chen, Juyang Wang.

**Project administration:** Huijuan Yuan.

**Software:** Juyang Wang, Dou Ying.

**Supervision:** Xue Yang, Dou Ying.

**Validation:** Xue Yang.

**Visualization:** Yangshiyu Li, Dou Ying.

**Writing – original draft:** Yiqi Chen, Huijuan Yuan.

**Writing – review & editing:** Xue Yang, Juyang Wang, Yangshiyu Li, Dou Ying.

## Supplementary Material

Supplemental Digital Content
