# Supplementary material for: Weight loss increases all-cause mortality in overweight or obese patients with diabetes: A meta-analysis
Source: Medicine (Baltimore). 2018 Aug 21;97(35):e12075. doi: 10.1097/MD.0000000000012075 (PMC6392938; doi:10.1097/MD.0000000000012075)
Supplement: Supplemental Digital Content [file medi-97-e12075-s001.docx]

**Appendix 1. List of excluded studies and exclusion reason.**

| Exclusion reason |  |
| --- | --- |
| BMI in childhood, adolescence, early adulthood | [1,2,3] and so on |
| Surgery | [4,5,6,7,8,9,10,11,12,13,14,15,16,17,18] and so on |
| Review and meta-analysis | [19,20,21,22,23,24,25] and so on |
| Rationale and design | [26,43] and so on |
| Diabetic complication | [27] and so on |
| Not relevant data | [28,29,30,31,32,33,34,35,36,37] and so on |
| Patients | [38,39,40,41,42,43] and so on |
| Not relevant outcome | [44,45,46,47] and so on |
|  |  |
|  |  |
|  |  |
|  |  |
|  |  |
|  |  |
|  |  |
|  |  |
|  |  |
|  |  |
|  |  |
|  |  |
|  |  |
|  |  |
|  |  |
|  |  |

**References:**

1. Brambilla P, Lissau I, Flodmark CE, Moreno LA, Widhalm K, et al. (2007) Metabolic risk-factor clustering estimation in children: to draw a line across pediatric metabolic syndrome. Int J Obes (Lond) 31: 591-600.

2. Twig G, Afek A, Shamiss A, Derazne E, Landau RM, et al. (2014) Adolescence BMI and trends in adulthood mortality: a study of 2.16 million adolescents. J Clin Endocrinol Metab 99: 2095-2103.

3. Antwi F, Fazylova N, Garcon MC, Lopez L, Rubiano R, et al. (2012) The effectiveness of web-based programs on the reduction of childhood obesity in school-aged children: A systematic review. JBI Libr Syst Rev 10: 1-14.

4. Ells LJ, Mead E, Atkinson G, Corpeleijn E, Roberts K, et al. (2015) Surgery for the treatment of obesity in children and adolescents. Cochrane Database Syst Rev: D11740.

5. Iossa A, De Peppo F, Caccamo R, Watkins BM, Abbatini F, et al. (2017) Laparoscopic sleeve gastrectomy in adolescents with or without syndromic obesity: two years follow-up. Eat Weight Disord.

6. Mahdy T, Al WA, Schou C (2016) Efficacy of single anastomosis sleeve ileal (SASI) bypass for type-2 diabetic morbid obese patients: Gastric bipartition, a novel metabolic surgery procedure: A retrospective cohort study. Int J Surg 34: 28-34.

7. Lee WJ, Almulaifi A, Chong K, Yao WC, Tsou JJ, et al. (2016) Bariatric versus diabetes surgery after five years of follow up. Asian J Surg 39: 96-102.

8. Mingrone G, Panunzi S, De Gaetano A, Guidone C, Iaconelli A, et al. (2015) Bariatric-metabolic surgery versus conventional medical treatment in obese patients with type 2 diabetes: 5 year follow-up of an open-label, single-centre, randomised controlled trial. Lancet 386: 964-973.

9. Douglas IJ, Bhaskaran K, Batterham RL, Smeeth L (2015) Bariatric Surgery in the United Kingdom: A Cohort Study of Weight Loss and Clinical Outcomes in Routine Clinical Care. PLoS Med 12: e1001925.

10. Hsu CC, Almulaifi A, Chen JC, Ser KH, Chen SC, et al. (2015) Effect of Bariatric Surgery vs Medical Treatment on Type 2 Diabetes in Patients With Body Mass Index Lower Than 35: Five-Year Outcomes. JAMA Surg 150: 1117-1124.

11. Apovian CM, Huskey KW, Chiodi S, Hess DT, Schneider BE, et al. (2013) Patient factors associated with undergoing laparoscopic adjustable gastric banding vs Roux-en-Y gastric bypass for weight loss. J Am Coll Surg 217: 1118-1125.

12. Garciacaballero M, Navarrete S, Favretti F, Celik A, Del CD (2013) Diabetes surgery in type 2 BMI 24-29 vs IMC 30-34 diabetic patients: is there differences among restrictive, malabsorptive and gastric bypass procedures? Nutr Hosp 28 Suppl 2: 23-30.

13. Acquafresca PA, Palermo M, Duza GE, Blanco LA, Serra EE (2015) [Gastric Bypass versus Sleeve gastrectomy: comparison between type 2 Diabetes weight loss and complications. Review of randomized control trails]. Acta Gastroenterol Latinoam 45: 143-154.

14. Van Nieuwenhove Y, Spriet E, Sablon T, Van Daele E, Willaert W, et al. (2016) Metabolic surgery in patients over 60 years old: short- and long-term results. Acta Chir Belg 116: 362-366.

15. Yin J, Xu L, Mao Z, Zhou X, Zhu Z, et al. (2014) Laparoscopic Roux-en-Y gastric bypass for type 2 diabetes mellitus in nonobese Chinese patients. Surg Laparosc Endosc Percutan Tech 24: e200-e206.

16. Scopinaro N, Adami GF, Papadia FS, Camerini G, Carlini F, et al. (2014) Effects of gastric bypass on type 2 diabetes in patients with BMI 30 to 35. Obes Surg 24: 1036-1043.

17. Scheen AJ, De Flines J, De Roover A, Paquot N (2009) Bariatric surgery in patients with type 2 diabetes: benefits, risks, indications and perspectives. Diabetes Metab 35: 537-543.

18. Schauer PR, Mingrone G, Ikramuddin S, Wolfe B (2016) Clinical Outcomes of Metabolic Surgery: Efficacy of Glycemic Control, Weight Loss, and Remission of Diabetes. Diabetes Care 39: 902-911.

19. Simmonds M, Burch J, Llewellyn A, Griffiths C, Yang H, et al. (2015) The use of measures of obesity in childhood for predicting obesity and the development of obesity-related diseases in adulthood: a systematic review and meta-analysis. Health Technol Assess 19: 1-336.

20. Chuah LL, Papamargaritis D, Pillai D, Krishnamoorthy A, le Roux CW (2013) Morbidity and mortality of diabetes with surgery. Nutr Hosp 28 Suppl 2: 47-52.

21. Fujioka K (2010) Benefits of moderate weight loss in patients with type 2 diabetes. Diabetes Obes Metab 12: 186-194.

22. Liu XM, Liu YJ, Zhan J, He QQ (2015) Overweight, obesity and risk of all-cause and cardiovascular mortality in patients with type 2 diabetes mellitus: a dose-response meta-analysis of prospective cohort studies. Eur J Epidemiol 30: 35-45.

23. Aucott LS (2008) Influences of weight loss on long-term diabetes outcomes. Proc Nutr Soc 67: 54-59.

24. Bales CW, Buhr G (2008) Is obesity bad for older persons? A systematic review of the pros and cons of weight reduction in later life. J Am Med Dir Assoc 9: 302-312.

25. Schellenberg ES, Dryden DM, Vandermeer B, Ha C, Korownyk C (2013) Lifestyle interventions for patients with and at risk for type 2 diabetes: a systematic review and meta-analysis. Ann Intern Med 159: 543-551.

26. Htike ZZ, Yates T, Brady EM, Webb D, Gray LJ, et al. (2016) Rationale and design of the randomised controlled trial to assess the impact of liraglutide on cardiac function and structure in young adults with type 2 diabetes (the LYDIA study). Cardiovasc Diabetol 15: 102.

27. Docherty NG, Canney AL, le Roux CW (2015) Weight loss interventions and progression of diabetic kidney disease. Curr Diab Rep 15: 55.

28. Dutton GR, Lewis CE (2015) The Look AHEAD Trial: Implications for Lifestyle Intervention in Type 2 Diabetes Mellitus. Prog Cardiovasc Dis 58: 69-75.

29. Iepsen EW, Torekov SS, Holst JJ (2014) Therapies for inter-relating diabetes and obesity - GLP-1 and obesity. Expert Opin Pharmacother 15: 2487-2500.

30. Wilding JP (2014) The importance of weight management in type 2 diabetes mellitus. Int J Clin Pract 68: 682-691.

31. Balducci S, Zanuso S, Cardelli P, Salvi L, Mazzitelli G, et al. (2012) Changes in physical fitness predict improvements in modifiable cardiovascular risk factors independently of body weight loss in subjects with type 2 diabetes participating in the Italian Diabetes and Exercise Study (IDES). Diabetes Care 35: 1347-1354.

32. Logue J, Walker JJ, Leese G, Lindsay R, McKnight J, et al. (2013) Association between BMI measured within a year after diagnosis of type 2 diabetes and mortality. Diabetes Care 36: 887-893.

33. Tseng CH (2013) Obesity paradox: differential effects on cancer and noncancer mortality in patients with type 2 diabetes mellitus. Atherosclerosis 226: 186-192.

34. Ross SA, Dzida G, Vora J, Khunti K, Kaiser M, et al. (2011) Impact of weight gain on outcomes in type 2 diabetes. Curr Med Res Opin 27: 1431-1438.

35. Colberg SR, Albright AL, Blissmer BJ, Braun B, Chasan-Taber L, et al. (2010) Exercise and type 2 diabetes: American College of Sports Medicine and the American Diabetes Association: joint position statement. Exercise and type 2 diabetes. Med Sci Sports Exerc 42: 2282-2303.

36. Gruber A, Nasser K, Smith R, Sharma JC, Thomson GA (2006) Diabetes prevention: is there more to it than lifestyle changes? Int J Clin Pract 60: 590-594.

37. Franco M, Bilal U, Ordunez P, Benet M, Morejon A, et al. (2013) Population-wide weight loss and regain in relation to diabetes burden and cardiovascular mortality in Cuba 1980-2010: repeated cross sectional surveys and ecological comparison of secular trends. BMJ 346: f1515.

38. Sorensen TI, Rissanen A, Korkeila M, Kaprio J (2005) Intention to lose weight, weight changes, and 18-y mortality in overweight individuals without co-morbidities. PLoS Med 2: e171.

39. Williamson DF, Pamuk E, Thun M, Flanders D, Byers T, et al. (1999) Prospective study of intentional weight loss and mortality in overweight white men aged 40-64 years. Am J Epidemiol 149: 491-503.

40. Campmans-Kuijpers MJ, Sluijs I, Nothlings U, Freisling H, Overvad K, et al. (2016) The association of substituting carbohydrates with total fat and different types of fatty acids with mortality and weight change among diabetes patients. Clin Nutr 35: 1096-1102.

41. Yaari S, Goldbourt U (1998) Voluntary and involuntary weight loss: associations with long term mortality in 9,228 middle-aged and elderly men. Am J Epidemiol 148: 546-555.

42. Droyvold WB, Lund NT, Lydersen S, Midthjell K, Nilsson PM, et al. (2005) Weight change and mortality: the Nord-Trondelag Health Study. J Intern Med 257: 338-345.

43. MacLaughlin HL, Hall WL, Condry J, Sanders TA, Macdougall IC (2015) Participation in a Structured Weight Loss Program and All-Cause Mortality and Cardiovascular Morbidity in Obese Patients With Chronic Kidney Disease. J Ren Nutr 25: 472-479.

44. Amador LF, Al SS, Markides KS, Goodwin JS (2006) Weight change and mortality among older Mexican Americans. Aging Clin Exp Res 18: 196-204.

45. Liu XM, Liu YJ, Zhan J, He QQ (2015) Overweight, obesity and risk of all-cause and cardiovascular mortality in patients with type 2 diabetes mellitus: a dose-response meta-analysis of prospective cohort studies. Eur J Epidemiol 30: 35-45.

46. de Mutsert R, Sun Q, Willett WC, Hu FB, van Dam RM (2014) Overweight in early adulthood, adult weight change, and risk of type 2 diabetes, cardiovascular diseases, and certain cancers in men: a cohort study. Am J Epidemiol 179: 1353-1365.

47. Mulnier HE, Seaman HE, Raleigh VS, Soedamah-Muthu SS, Colhoun HM, et al. (2006) Mortality in people with type 2 diabetes in the UK. Diabet Med 23: 516-521.
